# Effects of Hormonal Contraceptives on Mood: A Focus on Emotion Recognition and Reactivity, Reward Processing, and Stress Response

**DOI:** 10.1007/s11920-019-1095-z

**Published:** 2019-11-07

**Authors:** Carolin A. Lewis, Ann-Christin S. Kimmig, Rachel G. Zsido, Alexander Jank, Birgit Derntl, Julia Sacher

**Affiliations:** 1 Emotion Neuroimaging Lab, Max Planck Institute for Human Cognitive and Brain Sciences, Stephanstr. 1A, 04103 Leipzig, Germany; 2 International Max Planck Research School on Neuroscience of Communication: Function, Structure, and Plasticity, Leipzig, Germany; 3 Department of Psychiatry and Psychotherapy, University of Tuebingen, Calwerstr, 14, 72076 Tuebingen, Germany; 4 International Max Planck Research School for Cognitive and Systems Neuroscience, University of Tuebingen, Tuebingen, Germany; 5 Department of Neurology, Max Planck Institute for Human Cognitive and Brain Sciences, Leipzig, Germany; 6 Department of Obstetrics, University Hospital Leipzig, Leipzig, Germany; 7 Werner Reichardt Center for Integrative Neuroscience, University of Tuebingen, Tuebingen, Germany; 8 LEAD Research School and Graduate Network, University of Tuebingen, Tuebingen, Germany; 9 Clinic for Cognitive Neurology, University of Leipzig, Leipzig, Germany

**Keywords:** Hormonal contraceptives, Mood, Depression, Emotion, Reward, Stress

## Abstract

**Purpose of Review:**

We review recent research investigating the relationship of hormonal contraceptives and mood with a focus on relevant underlying mechanisms, such as emotion recognition and reactivity, reward processing, and stress response.

**Recent Findings:**

Adverse effects of hormonal contraceptives (HCs) on mood seem most consistent in women with a history of depressive symptoms and/or previous negative experience with HC-intake. Current evidence supports a negativity bias in emotion recognition and reactivity in HC-users, although inconsistent to some extent. Some data, however, do indicate a trend towards a blunted reward response and a potential dysregulation of the stress response in some HC-users.

**Summary:**

HC-effects on psychological and neurophysiological mechanisms underlying mood are likely context-dependent. We provide suggestions on how to address some of the contributing factors to this variability in future studies, such as HC-dose, timing, administration-mode, and individual risk. A better understanding of how and when HCs affect mood is critical to provide adequate contraceptive choices to women worldwide.

## Introduction

With currently more than 100 million users worldwide [1], hormonal contraceptives (HCs) represent one of the most influential discoveries of the twentieth century [2]. HCs provide an effective option for contraception and safe family planning as well as for managing cycle-related physiological symptoms (e.g., ovulation pain, acne, hirsutism). Although this suggests that HC-use is beneficial for many women, there is a subset of women who suffer severe mood-related side effects. Thus, while substantial research has been dedicated to the physiological consequences of HC-use, such as cardiovascular risk, few studies have investigated the effects of HCs on mood and behavior.

**Fig. 1:**
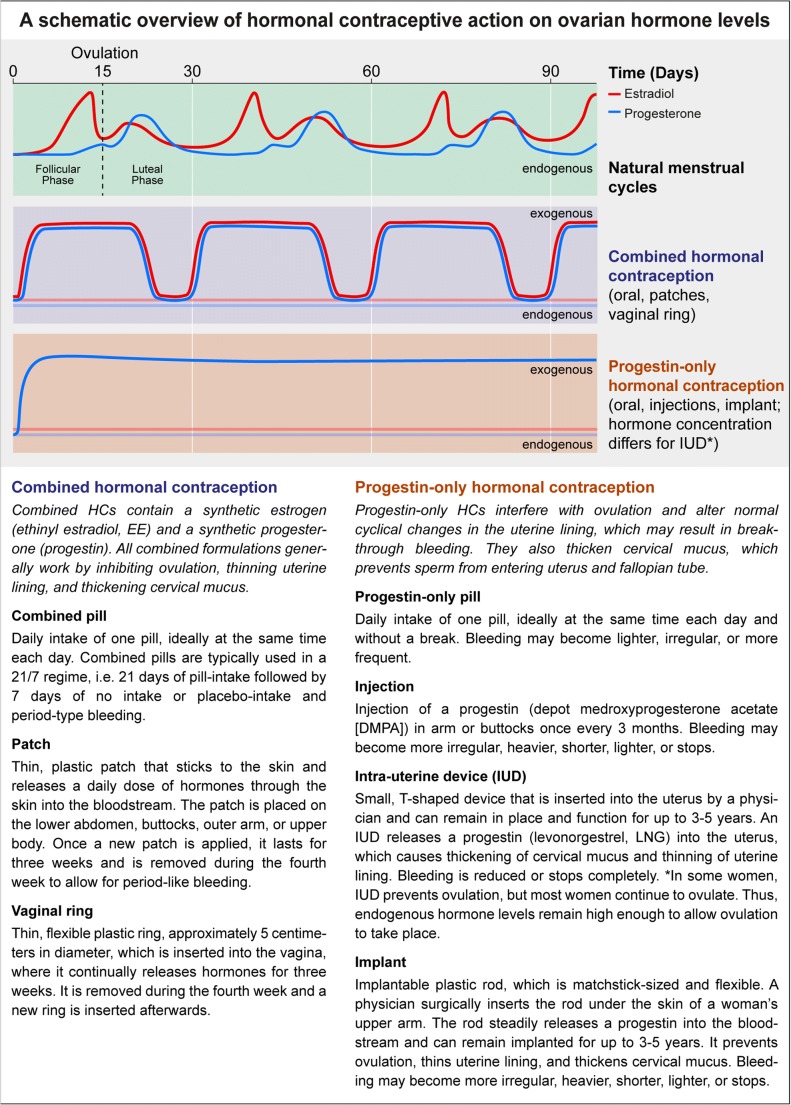
Comparison of ovarian hormone profiles across the natural menstrual cycle (top row), and during intake of most common hormonal contraceptives, such as combined hormonal contraception (middle row), and progestin-only hormonal contraception (bottom row). The modes of action as well as intake characteristics of the most common hormonal contraceptives are described below.

Given that side effects such as depressive symptoms are typically reported as the main reason for discontinuing HC-use [3, 4] and the relative scarcity of neuroimaging studies currently published in this area, additional research efforts to shed light on the neuropsychological side effects of HCs are warranted. With the emerging field of reproductive neuroscience, scientists are beginning to investigate the neural effects of HC-use in humans. A better understanding of how HC-use influences mood may have a critical impact on translational psychiatry, considering that women are approximately twice as likely as men to develop depression [5] and ovarian hormonal fluctuations have been associated with depression susceptibility and prevalence in women [6]. Epidemiological data suggest that hormonal transition periods across the female lifespan, such as puberty, pregnancy and postpartum, and the perimenopause, are windows of heightened risk to develop depression [7•], comprising a possible reproductive subtype of depression [8]. Certain women are particularly susceptible to the subtle hormone fluctuations across the menstrual cycle, which may result in the development of premenstrual dysphoric disorder (PMDD) [9]. Given these reported associations between hormone fluctuations and depression susceptibility, and that HCs introduce synthetic ovarian hormones thereby modulating endogenous ovarian hormone production (for overview, see Fig. 1 and [10, 11, 89, 90]), we review recent research investigating the relationship of HC-use and mood with a focus on relevant underlying mechanisms, such as emotion recognition and reactivity, reward processing, and stress responsivity.

Relying on Danish Registry data, Skovlund and colleagues [12••] recently reported a link between antidepressant prescription and HC-use. The authors included data from more than one million women in the age of 15–34 years, who were using combined estradiol/progestin as well as progestin-only HCs in all available forms of administration (see Fig. 1 and [10, 11, 89, 90] for an overview of HC methods). In those women, risk ratios for first diagnoses of depression or first antidepressant-use increased during the first 6 months after initiation of HC-use (1.8-fold relative risk compared with naturally cycling women). Similarly, Zettermark and colleagues [13••] investigated the prescription of psychotropic drugs (anxiolytics, hypnotics, sedatives, or antidepressants) within the first year of HC-use in a sample of 800,000 women from a Swedish health registry. Reported rates for psychotropic drug use indicated an adjusted odds ratio of 1.34 for a first-time psychotropic drug prescription in HC-users. However, both studies [12••, 13••] were correlational in nature and reporting relative risks can be misleading as the incidence of these events is quite low [14]. While causation is not determinable in observational designs, both studies [12••, 13••] investigated impressive sample sizes, providing essential epidemiological evidence to develop hypotheses for potential mechanisms underlying the reported associations of HC-use and depression risk.

Randomized, placebo-controlled trials (RCTs) represent the gold standard in intervention-based studies, in that they can provide the strongest possible evidence for causal effects. Several groups have now successfully applied this study design to investigate HC-effects on mood. Zethraeus and colleagues [15••] included over 300 women in a double-blind RCT, testing the effect of a combined oral contraceptive (OC) versus placebo, on well-being and mood. Over the course of 3 months, women in the OC group reported significantly lower global scores on self-reported well-being compared with placebo, driven by the negative effect of OCs on scales measuring positive well-being, self-control, and vitality. However, mean depression scores did not differ significantly across groups and time points in self-reported Beck Depression Inventory (BDI) scores.

Another Swedish group took a more unconventional approach in their double-blind RCT: they aimed to sample participants more representative of HC-users in the general population, thus deciding not to exclude women with previous or ongoing psychiatric disorders and respective medication, nor any women with a history of OC-use-associated onset of depressed mood [16••, 17••]. In total, over 200 women participated in either a placebo or combined OC group for three treatment cycles. The authors reported small but significant mood-related adverse effects of OCs in self-reported anxiety, irritability, and mood swings. No significant effects of OCs were observed for the Montgomery-Asberg Depression Rating Scale. However, some women in the OC group also reported improvements in mood during the premenstrual phase of the cycle. Women with previous negative OC-associated experiences reported significantly more severe depressed mood after completion of the 3-month trial compared with women with no such history. A further aspect to consider is the effect of HCs on the expression of premenstrual mood symptoms. Here, one study reports no effect of HC-use on premenstrual mood (using a prospective cross-over design; [18]), while another study supports a beneficial association between HC-use and premenstrual mood symptoms (although cross-sectional; [19]).

In summary, the data currently available supports some mood-related side effects of HC-use, most convincingly shown in women with a history of depressive symptoms. However, some women may experience beneficial effects of HC-use, specifically on premenstrual mood symptoms (see [20] for review). As HC-related side effects on mood are not fully understood to date, additional research efforts to shed light on a possible impact of HCs on the mechanisms underlying mood regulation are warranted. Therefore, we review recent research on HC-effects on main psychological and neurophysiological mechanisms underlying mood regulation, such as the behavioral and neural correlates of emotion recognition and reactivity, reward processing, and stress response (Table 1).

**Table 1:** Overview of results from studies investigating HC-effects on psychological and neurophysiological mechanisms underlying mood regulation—emotion recognition and reactivity, reward processing, and stress response. Studies are listed in order of appearance in the text

	Research design	Sample size and HC method	Research modality	Task	Results		Main findings in HC-users
**Emotion recognition**							
Pahnke et al. [21]	Cross-sectional	42 combined OC 53 NC (35 follicular, 18 luteal)	Behavioral	RMET	OC-users performed significantly worse in complex face recognition independent of emotional valence or type of OC (androgenic vs antiandrogenic).	↓	Impaired emotion recognition
Hamstra et al. [22]	Cross-sectional	49 combined OC 44 NC (21 early follicular, 23 luteal)	Behavior-genotype interaction	Facial expression recognition task, RMET	OC-users with MC haplotype 1 and 3 performed generally worse in face recognition task than luteal NC women (trend-level). OC-users with MC haplotype 2 recognized less positive characteristics in the RMET than luteal NC women.	↓	Impaired emotion recognition
Hamstra et al. [23]	Cross-sectional	44 combined OC 40 NC (11 follicular, 29 luteal)	Behavior-genotype interaction	Facial expression recognition task, emotional categorization, and memory task	OC-users showed worse recognition of anger independent of MC haplotype.	↓	Impaired emotion recognition of negative emotions
					OC-users with MC haplotype 1 and 3 recognized fearful and sad images significantly better and recalled more negative characteristics, but also had longer reaction times for detecting these emotions.	↑	Attention bias to negative emotions in MC haplotype 1 and 3 carriers
Hamstra et al. [24]	Cross-sectional	26 OC^1^ 14 follicular NC	Behavioral	Facial expression recognition task	OC-users showed worse recognition of facial expressions depicting anger, as well as a trend for sadness and disgust, compared with NC women.	↓	Impaired emotion recognition of negative emotions
Hamstra et al. [25]	Longitudinal	57 combined OC (within subjects active vs. inactive pill phase) 39 NC (within subjects early follicular vs. luteal)	Behavior-genotype interaction	Facial expression recognition task, RMET	OC-users showed worse recognition of sadness and happiness (trend-level) and had shorter reaction times for detecting anger and happiness. OC-users recognized more positive characteristics in RMET	↔	Mixed findings: both impaired and enhanced emotion recognition
Radke et al. [26•]	Cross-sectional	25 combined OC (inactive phase) 30 combined OC (active phase) 18 NC	Behavioral	Affective responsiveness task, emotion recognition task, perspective-taking task	No differences in emotion recognition and perspective taking between OC-users and NC women.	↔	No differences in emotion recognition
					Increased accuracy for OC-users in active phase in affective responsiveness compared with OC-users in an inactive phase.	↑	Enhanced emotional reactivity in active OC phase
**Emotional reactivity**							
Spalek et al. [27•]	Cross-sectional	1215 HC^1^ (majority OC) 954 NC	Behavioral	Picture rating task, picture memory task	HC-users rated emotional pictures (negative and positive) more emotionally intense and neutral images less arousing than NC women.	↑	Enhanced emotional reactivity
					HC-users remembered significantly more emotional pictures (positive and negative) than NC women which were mediated by valence/arousal ratings.	↑	Enhanced emotional memory
Gingnell et al. [28]	RCT	17 combined OC-starters 17 placebo-starters (both groups with previous mood-related side effects of OCs)	Task fMRI	Emotional face-matching task	No differences in face-matching accuracy.	↔	Similar ratings
					OC-starters had significantly more mood swings and depressed mood after 1 month of intake compared with pre-start and to the placebo group.	↑	Mood swings and depressed mood
					OC-starters had reduced BOLD response in the left insula, left MFG, and bilateral IFG compared with placebo and reduced BOLD response in bilateral IFG compared with pre-start.	↓	Blunted emotional reactivity
					Decreased habituation of amygdala in OC-starters compared with placebo between time points.	↑	Higher vigilance for negative emotional stimuli
Miedl et al. [29•]	Cross-sectional	23 combined OC 30 NC	Task fMRI	Traumatic vs neutral video clips	No differences in valence and arousal ratings.	↔	Similar ratings
					Enhanced BOLD responses in OC-users in the insula and dorsal ACC during traumatic vs. neutral clip viewing.	↑	Enhanced emotional reactivity for traumatic content
Merz et al. [30]	Cross-sectional	29 combined OC 30 luteal NC 39 men	Task fMRI, physiology	Fear acquisition and extinction	Slower habituation of SCR rates, correlated with increased BOLD signal in response to fear-evoking stimuli in OC-users compared with NC women in the right amygdala, right ACC, bilateral thalamus, and vmPFC.	↔ ↑	Similar emotional reactivity in the acquisition phase Increased emotional reactivity in the extinction phase
Hwang et al. [31]	Cross-sectional	16 combined OC 32 NC (16 high estradiol, 16 low estradiol) 37 men	Task fMRI	Fear conditioning, extinction and recall procedures	Reduced BOLD response during fear conditioning in the insular cortex, MCC, amygdala, and hypothalamus in OC-users compared with high estradiol NC women.	↓	Blunted emotional reactivity during fear conditioning
					No differences between OC-users and NC women for unconditioned fear, fear extinction, and recall.	↔	Similar emotional reactivity in fear extinction and recall
Armbruster et al. [32]	Cross-sectional	35 combined OC 35 NC (within-subjects early follicular vs. late luteal)	Physiology (SCR, startle magnitude)	Acoustic startle response task during image presentation	No difference in valence and arousal ratings between OC-users and NC women.	↔	Similar ratings
					OC had blunted startle magnitudes and SCR, especially for negative images.	↓	Blunted emotional reactivity
**Reward processing**							
Petersen et al. [33]	Cross-sectional	44 combined OC 46 NC	Structural MRI	–	OC-use associated with lower cortical thickness in lateral OFC and posterior cingulate cortex.	↓	Lower cortical thickness in reward-related region
Bonenberger et al. [34]	Cross-sectional	12 OC^1^ 12 NC	Task fMRI	Monetary incentive task	Enhanced BOLD response during monetary reward expectation in anterior insula and inferior PFC in OC-users compared with NC women in the follicular phase.	↑	Enhanced reward response
Arnoni-Bauer et al. [35]	Cross-sectional	12 combined OC 20 NC	Task fMRI	Visual food cues	OC-users show similar BOLD response as NC women in the luteal phase but greater BOLD response as NC women in the follicular phase in reward response (amygdala, putamen) and decision-making regions (PFC).	↔↑	Similar to enhanced reward response
Scheele et al. [36]	Cross-sectional	21 HC (16 combined OC, 5 IUS) 19 NC	Task fMRI	Attractiveness rating under single oxytocin nasal dose	Oxytocin increased attractiveness ratings of the partner’s face in NC women but not in HC-users. Reduced BOLD response in striatal reward regions (NAcc, VTA) in OC-users compared with NC women.	↓	Blunted reward response
Jakob et al. [37•]	Longitudinal	38 combined OC (within-subjects active vs. inactive pill phase) 30 NC (within-subjects early vs. late follicular)	Behavior-hormone-genotype interaction	Probabilistic reinforcement learning task	Decrease in ability to avoid punishment with rising estradiol levels in 9RP carriers NC women, no such behavioral variations in OC-users according to DAT1-genotype differences or intake phase.	↔	No genotype interaction for reward responses
**Stress response**							
Merz et al. [38]	Cross-sectional	30 combined OC 60 NC	Behavioral, physiological	SECPT	Blunted cortisol response in OC-users compared with NC women after stress exposure.	↓	Blunted stress response
Barel et al. [39]	Cross-sectional	20 combined OC 17 NC	Behavioral, physiological	TSST	Blunted cortisol response in OC-users compared with NC women after stress exposure.	↓	Blunted stress response
Nielsen et al. [40]	Cross-sectional	49 combined OC 60 NC	Behavioral, physiological	Emotional recall after CPS	Blunted cortisol after stress exposure, paralleled by weaker performance in emotional recall response in OC-users compared with NC women.	↓	Blunted stress response
Mordecai et al. [41]	Longitudinal	39 combined OC (within-subjects active vs. inactive pill phase) 40 NC (within-subjects follicular vs. luteal)	Behavioral, physiological	Emotional recall after TSST	Blunted cortisol response and worse emotional recall for negative words in OC-users (similar for both active and inactive pill phase) compared with NC women after stress exposure.	↓	Blunted stress response
					Higher baseline cortisol levels in OC-users compared with NC women.	↑	Higher baseline cortisol
Hertel et al. [42••]	Cross-sectional	74 OC (70 combined OC, 4 progestin-only) 159 NC	Structural MRI, physiological	–	Higher baseline cortisol levels and reduced hippocampal gray matter in OC-users compared with NC women.	↑ ↓	Higher baseline cortisol Reduced hippocampal volume

^1^No information stated on which specific HC/OC-type used in the study

*ACC* anterior cingulate cortex, *BOLD* blood oxygen level-dependent, *CPS* cold-pressor stress, *DAT-1* dopamine transporter, *fMRI* functional magnetic resonance imaging, *HC* hormonal contraceptive, *IFG* inferior frontal gyrus, *MC* mineralocorticoid, *MCC* middle cingulate cortex, *MFG* middle frontal gyrus, *NAcc* nucleus accumbens, *NC* naturally cycling, *OC* oral contraceptive, *OFC* orbitofrontal cortex, *PFC* prefrontal cortex, *RCT* randomized, placebo-controlled trial, *RMET* Reading the Eyes in the Mind Test, *SECPT* socially evaluated cold-pressor test, *SCR* skin conductance response, *TSST* Trier social stress test, *vmPFC* ventromedial prefrontal cortex, *VTA* ventral tegmental area

## Influence of HCs on Psychological and Neurophysiological Mechanisms Underlying Mood Regulation

### Emotion Recognition and Reactivity

Negativity biases in key facets of emotion processing such as emotion recognition and emotional reactivity are thought to substantially contribute to the development and maintenance of depressed mood [43]. Mitigating negativity biases in emotion recognition and reducing emotional reactivity to negative stimuli can be effective strategies to improve mood [43–45].

The ability to correctly recognize emotional content from faces represents one major component of nonverbal communication [46], and impairments in this ability may play an important role in the development and maintenance of depressive symptoms [47, 48]. Several studies found impaired emotion recognition in OC-users [21, 22], particularly for negative emotions [23–25], compared with naturally cycling women. For example, Pahnke and colleagues [21] report overall facial emotion recognition deficits in OC-users independent of emotional valence during the Reading-the-Mind-in-the-Eyes task, whereas Hamstra and colleagues [23, 24] identified a negativity bias in emotion recognition and emotional memory during a facial expression recognition task and an emotional categorization and memory task, respectively. Here, OC-users had significantly lower recognition accuracies for angry faces compared with naturally cycling women [23, 24]. The authors further suggest that OC-users who are carriers of the mineralocorticoid receptor (MR) haplotype 1 or 3 have a more pronounced negativity bias, as these OC-users (1) had higher accuracy rates for detecting fearful and sad faces (unlike for angry faces), (2) had significantly longer reaction times for detecting these negative emotions, and (3) had better recall of negative characteristics in an emotional memory task, thus implicating an attention bias towards negative emotions [23]. Therefore, MR haplotype 1 or 3 carriers might be more vulnerable to depressogenic side effects of OCs than MR haplotype 2 carriers. Contrary to these findings, Radke and Derntl [26•] did not find evidence for an emotion recognition deficit in OC-users compared with naturally cycling women; however, they used only high-intensity emotional faces.

The current literature seems to confirm a negativity bias in emotion recognition in HC-users, i.e., deficits in recognizing emotions accurately [21, 22, 25] as well as an attentional bias to negative emotions [23–25]. However, emotion recognition abilities in HC-users seem to be affected by the task used in the study [25, 26•] or individual (epi-)genetic characteristics [22, 25, 23].

In addition to emotion recognition, emotional reactivity may also be linked to depressive symptoms. Emotional reactivity is the emotional response to an event, which can occur through multiple systems and differs in intensity and duration between individuals [49]. More intense and labile emotions, often accompanied by physiological arousal [50], have been associated with more depressive and internalizing symptoms [51, 52]. While Radke and Derntl [26•] did not observe any differences in emotion recognition between OC-users and naturally cycling women, they reported that OC-users during the active OC-intake phase performed significantly better in an emotional reactivity task (affective responsiveness task) than OC-users during the pill-free week. Therefore, the active intake of OCs seems to be linked to an enhanced emotional reactivity towards positive as well as negative emotional scenarios. In line with these findings, a large-scale study recently showed that women using HCs showed significantly higher emotional reactivity by rating the valence of emotional stimuli more emotionally intense and recalling these emotional pictures significantly better than did naturally cycling women [27•].

Neuroimaging research sheds further light on the possible modulatory effects of HCs on emotional reactivity. In a double-blind, placebo-controlled, functional magnetic resonance imaging (fMRI) study that only included women who had previously experienced OC-induced depressogenic side effects, Gingnell and colleagues [28] observed no behavioral differences between the OC-assigned group and the placebo-assigned group in an emotional reactivity task (face-matching task with only negative faces) after 1 month of intake. The OC group did, however, show decreased habituation of the amygdala blood oxygenation level-dependent (BOLD) response compared with the placebo group. This finding could point towards a higher continued vigilance for negative emotional stimuli and therefore a biased attention towards negative stimuli in OC-users, possibly explaining adverse effects on mood. The OC group also showed reduced BOLD response of the left insula, the left middle frontal gyrus, and the bilateral inferior frontal gyri compared with the placebo group in response to negative emotional face stimuli [28]. However, these differences in BOLD response occurred in brain regions that are otherwise activated for positive or salient emotional stimuli.

Specifically for fear processing, OC-induced effects on neural activation have been reported, such as enhanced activation of the fear network in OC-users compared with naturally cycling women, particularly in the insula and the dorsal anterior cingulate cortex (ACC) [29•]. These activation differences were independent of the valence and arousal ratings of the presented traumatic videos, which were similar between groups. Consistent with these findings, another study [30] found increased emotional arousal indicated by slower habituation of skin conductance response (SCR) rates, a physiological measure of the autonomic stress response, to be correlated with an increased BOLD signal in response to fear-evoking stimuli in OC-users compared with naturally cycling women. Group differences occurred in the right amygdala, right ACC, bilateral thalamus, and ventromedial prefrontal cortex (vmPFC). Unlike the previous study, Hwang and colleagues [31] did not observe any differences between OC-users and naturally cycling women in fear extinction but reported group differences for fear conditioning, i.e., reduced BOLD response of the fear network in OC-users compared with naturally cycling women. These neural correlates are further supported by physiological data, namely blunted SCR and startle reflex during fear conditioning in OC-users compared with naturally cycling women [32]. While emotional reactivity seems to be enhanced during fear extinction [30], neural [31] as well as physiological responses [32] are reduced during fear conditioning in OC-users.

Overall, the current data suggests a negativity bias in emotional reactivity shown by reduced BOLD responses to negative stimuli in brain regions that are otherwise relevant for processing salient and positive emotions [28], and enhanced BOLD responses in brain regions relevant for processing negative emotions, such as fear [29•, 30]. These neuroimaging results are often not paralleled by behavioral outcomes [26•, 28, 29•, 32] and thus need to be interpreted with caution. However, as emotional reactivity occurs by definition through multiple systems [49], it might as well be that HC-use specifically impacts a very early stage of emotion processing, as reflected by HC-induced modulation of emotional reactivity networks in the brain. On this account, further experimental designs including psychological, physiological, and neuroimaging measures when investigating HC-effects on emotional reactivity are highly encouraged.

### Reward Processing

Recent models from computational psychiatry propose that negative mood may reflect the cumulative impact of differences between reward outcomes and expectations (e.g., [53, 54]). These models suggest a bidirectional interaction between mood and reward processing, which likely plays an important adaptive role in healthy behavior or, if compromised, could contribute to depressive disorders via a blunted hedonic response to rewards, i.e., anhedonia [55].

On a neural level, both endogenous estradiol and progesterone have neuroregulatory effects on the mesolimbic dopaminergic reward system [56–59]. In association with ovarian hormone fluctuations, changes in neural activation occur in the reward system [58], specifically in brain regions relevant for coding reward value and reward-expectancy such as the amygdala, the orbitofrontal cortex (OFC), and the striatum [60].

Literature on HC-related modulations of the reward system is relatively sparse. Petersen and colleagues [33] reported OC-use to be associated with significantly lower cortical thickness in the posterior cingulate cortex and the lateral OFC, with the latter revealing the most pronounced difference in cortical thickness between naturally cycling women and OC-users. This frontal cortex region is critical for the cognitive control of behavior, including response inhibition to stimuli with changing reward value [61]. Post hoc analyses suggest that these differences in cortical thickness were greater comparing OC-users and women in the follicular phase than comparing OC-users and women in the luteal phase. Yet, as this study used a cross-sectional design, we cannot infer causality nor establish a time-dependent association of OC-intake and OFC cortical thickness thus far.

OC-induced changes in brain morphology do not allow direct assumptions about behavioral changes, but task-based fMRI studies can shed light on potential behavioral consequences. In a comparison of naturally cycling women with OC-users during a monetary incentive task, OC-users were more sensitive to monetary rewards and showed enhanced BOLD response during monetary reward expectation in the anterior insula and inferior prefrontal cortex (PFC) relative to naturally cycling women in the follicular phase [34]. Another study observed greater neural activation to visual food stimuli in OC-users than naturally cycling women during the follicular phase, but no group differences between OC-users and naturally cycling women during the luteal phase [35]. This difference in BOLD response during the follicular phase was observed in brain regions of the reward system (amygdala, putamen) as well as executive frontal areas (PFC). The authors proposed that comparable progesterone levels in OC-users and naturally cycling women in the luteal phase may underlie the similar BOLD responses between groups (similar to [33]). However, these studies were limited by their cross-sectional design and small sample sizes [34, 35] or lack of behavioral outcome measures [35]. Another study [36] included behavioral outcome measures and reported enhanced attractiveness ratings of the partner’s face in naturally cycling women but not in HC-users after intranasal administration of oxytocin. The concomitant increased BOLD responses in nucleus accumbens (NAcc) and ventral tegmental area (VTA) were also more pronounced in the naturally cycling group than in the HC group. Taken together, task-based fMRI studies seem to provide rather mixed results, which could be due to the varying tasks used, e.g., investigating primary [35, 36] or secondary rewards [34]. Replication studies, preferably studies comparing performance in both primary and secondary reward tasks, are needed to further elucidate this issue.

Preclinical evidence suggests that endogenous estradiol levels can increase dopamine release in the reward system, specifically in the striatum [62, 63]. Behavioral studies in humans partly support this finding as a positive correlation between endogenous estradiol levels and enhanced reward sensitivity in women, but paradoxically no increase in motivation for higher rewards from the early to the late follicular phase (i.e., with rising endogenous estradiol levels) have been reported [64]. Women have also been shown to be less sensitive for immediate rewards with rising estradiol levels from the early to the late follicular phase, but this effect was mainly driven by women with lower frontal dopamine levels (based on the COMT Met158Val polymorphism) [65]. These results nurtured the hypothesis of a hormone-genotype interaction, suggesting that particularly women with lower dopamine distribution would be affected by endogenous estradiol changes. Jakob and colleagues [37•] tested this hypothesis and investigated how endogenous estradiol levels and polymorphisms of the dopamine transporter (DAT1) interact. In this study, women performed a probabilistic feedback learning task twice: naturally cycling women once during the early (low estradiol) and subsequently during the late follicular phase (high estradiol) in comparison with OC-users once during active and once during inactive pill phase. Results indicated a significant effect of DAT1-genotype on reinforcement learning in naturally cycling women only, i.e., a decrease in the ability to avoid punishment with rising estradiol levels in 9RP carriers. The OC group did not show any such behavioral variations according to DAT1-genotype differences or intake phase. While these results suggest a small, dopamine-agonistic effect of endogenous estradiol on reward and punishment sensitivity (see [66] for an overview), the influence of HC-induced changes in endogenous and exogenous estradiol levels on dopamine neurotransmission needs further research.

Overall, results from studies investigating the impact of HCs on reward processing are mixed (see Table 1): Studies have reported women on HCs to be more sensitive to rewards [34], to show comparable reward responses to naturally cycling women [35], or to experience blunted reward responses than naturally cycling women [33, 36, 37•] as well as lower cortical thickness in brain regions of the reward system [33]. Based on the evidence currently available, the hypothesis of a blunted reward response in HC-users compared with naturally cycling women appears most supported, but remains to be systematically investigated.

### Stress Responsivity

In women, high endogenous estradiol levels have been associated with an acutely blunted cortisol response, which is typically viewed as protective against acute psychosocial stress [67•]. A chronically blunted cortisol response, however, might increase the risk for depression. Atypical depression is characterized by hypoactivation of the hypothalamic-pituitary-adrenocortical (HPA) axis and describes a distinct pathophysiological phenotype, which is particularly common in women [6]. Recent work on the role of estradiol in the neural stress circuitry in women revealed increased BOLD response in the amygdala, hippocampus, and hypothalamus after a visual stress challenge in low endogenous estradiol states compared with high endogenous estradiol states (within-subject design, [68]). Notably, only healthy women demonstrated this endocrine regulation, while there was no evidence for this regulatory effect in women with recurrent depression in remission. This suggests a possible endocrine dysregulation associated with an altered stress response in women with depression (see also [69] for review).

HC-studies on stress responsivity using well-validated stress tasks consistently report a blunted cortisol response in OC-using women compared with naturally cycling women [38, 39]. Nielsen and colleagues also found a blunted cortisol response in OC-users compared with naturally cycling women, paralleled by weaker performance for memorizing an emotional story: While naturally cycling women in the stress condition had enhanced recall for gist and detail, OC-users did not show such effects on memory [40]. Another study [41] extended these findings by showing that the blunted cortisol response previously reported in OC-users is similar during both the active and the inactive pill phase, following a psychosocial stress test (Trier social stress test, TSST). Interestingly, the authors also observed that OC-users had higher baseline salivary cortisol levels than naturally cycling women. Another study further substantiated this finding by investigating OC-related alterations in the HPA axis in OC-users compared with naturally cycling women [42••]: The authors found overall elevated cortisol levels in OC-users as well as reduced hippocampal gray matter when investigating structural MRI scans. Given the evidence connecting chronic stress, elevated cortisol levels, and decreased hippocampal volume (e.g., [70]), these findings may indicate a potential protective effect of fluctuating endogenous estradiol levels through the mitigation of neurodegenerative effects of chronic stress on the hippocampus (see [67•] for review). The authors did not, however, find an association between cortisol levels and depressive symptoms (BDI scores) in OC-using women [42••]. Thus, the link between HC-use, chronic stress, and depression susceptibility warrants further investigation [71].

Taken together, HC-intake seems to chronically alter HPA axis regulation, mirrored by (a) blunted cortisol responses after acute psychosocial and physical stressors [38–41] and (b) elevated baseline cortisol levels [41, 42••] (see Table 1). Further research is required to systematically address HC-effects on the response to acute and chronic stress in different states of endogenous and exogenous ovarian hormones in health and disease to conclusively answer the question whether HC-effects on the stress response underlie mood-related HC side effects in women at risk.

## Summary and Future Directions

In this review, we provide a summary of the most recent literature on HC-effects on women’s mood, with a specific emphasis on some of the psychological and neurophysiological mechanisms that could underlie mood-related side effects of HCs, which have been reported to occur in subgroups of women. We have reviewed the influence of HCs on emotion and reward processing as well as stress responsivity. We conclude that most of the reported results have yet to be replicated, thus no clear consensus can be reached based on these relatively heterogeneous datasets. From a methodological point of view, it is challenging to draw conclusions from neuroimaging results, which are not always paralleled by behavioral outcomes, and vice versa. Moreover, many neurobiological mechanisms are still not well understood. Given these limitations, most of the reported results have to be interpreted with caution, as the evidence is observational in most studies, and therefore, we cannot infer causality.

Many studies in this field only include women using OCs or women on different HC methods without stratification for each method. Consequently, the strongest conclusions can be drawn for OC-effects, as most of the available data include this HC method. Studies that did not stratify for different HC methods allow only for limited interpretation, as they contain different compounds and amounts of exogenous ovarian hormones, and also differ in the way of administration (see Fig. 1) and, thus, metabolization. Accumulating evidence [12••, 13••] suggests that different HC methods have divergent effects on mood: Non-oral HC methods (patch, vaginal ring, LNG-IUD) are more strongly associated with depression diagnosis or antidepressive treatment than OCs (combined OC and progestin-only OC). While these findings were correlational, some studies did take an interventional approach: Aleknaviciute and colleagues show that LNG-IUD induced sensitized HPA axis responsivity on both an acute and a chronic stress parameter, compared with women taking combined OCs or naturally cycling women [72•]. However, there was no difference in depression scores 6 months after LNG-IUD insertion, but this study did not include a control group [73]. Vaginal ring contraception did not significantly modulate mood scores after 6 months of use [74, 75], and a systematic review found that vaginal ring users reported less depressive mood, irritability, and emotional liability than combined OC-users [76]. Concerning progestin-only HCs, a recent systematic review found only minimal association between progestin-only methods and validated depression measures [77•]. In summary, a direct comparison of different HC methods, ideally using a RCT, would add critical evidence to the current debate about potential negative side effects of HCs on mood. Such a systematic investigation could also provide insight regarding a more refined neurobiological understanding of how OCs may affect mood compared with other hormonal methods. Finally, this type of research would also have great clinical relevance by informing clinical recommendations for or against a specific HC method for a particular woman (for example, based on previous depression history).

A major methodological aspect that must be addressed but is rarely discussed or oversimplified concerns the way most neuroimaging studies use HC-intake as a control variable for a low ovarian hormone state. This is only partly true. Indeed, the assessment of peripheral plasma levels of endogenous ovarian hormones, i.e., estradiol and progesterone, reveals low hormone levels. However, if a woman continuously takes exogenous ovarian hormones, either in an oral or non-oral route of administration, these exogenous ovarian hormones cross the blood-brain barrier [78, 79], a fact that should be considered in the interpretation of neuroimaging findings. Yet, this is only one aspect to consider: We do not yet know how a high exogenous and a low endogenous hormonal state interact, e.g., via feedback loops and cellular signaling, and how the net-effect of such an interaction can differ from a state of continuously fluctuating endogenous hormonal levels during the menstrual cycle. It is therefore challenging to interpret data from indirect neuroimaging modalities in vivo in the context of neurobiological mechanisms underlying the effects of HCs on behavior and brain function in women.

One technique that could provide essential insight into how HC-induced hormonal states (i.e., low endogenous but high exogenous) may directly influence such neurobiological mechanisms in the brain is positron emission tomography (PET). Radioligand PET studies allow for the visualization and in vivo quantification of a specific neurochemical target at a specific molecular site [80] and thus could clarify the neurochemical changes accompanying HC-use. We still require more tracer development dedicated to ovarian hormone receptors, but there are promising candidates. Ethinyl estradiol, the most commonly used synthetic estrogen in oral contraceptive formulations, is an estrogen receptor alpha (ERα) agonist [81]. The tracer 16α-[18F]fluoroestradiol-17β (FES) can be used to image ERα, although FES is so far mostly used in clinical practice to assess breast cancer [82, 83] and needs further investigation for suitability of ER imaging in the human brain. Two FES-PET studies in female rats [84, 85] only observed specific binding in brain regions with high ER density (i.e., pituitary gland and hypothalamus). One FES-PET study [86] in a small, healthy, post-menopausal sample of women (*n* = 7) also found significant uptake in the pituitary, as well as in white matter, but administration of an ER antagonist only successfully reduced FES in the pituitary. In a recent review on sex hormones and available PET radiotracers [87], authors conclude that FES could be useful for assessing ER density in ER-dense brain regions but encourage development of novel PET tracers with higher affinity for further research. Progesterone receptor imaging can be done using the tracers 21-[18F]fluoro-16α-ethyl-19-norprogesterone (FENP), 21-[18F]fluoro-16α,17α-[(R)-(1′-α-furylmethylidene)-dioxy]-19-norpregn-4-ene-3,20-dione (FFNP), and the more metabolically stable 4-[18F]Fluoropropyl-Tanaproget (FPTP) [88], although this study [88] was performed in female rats in non-brain areas (e.g., uterus and ovaries) and has yet to be studied in vivo in the human brain. Thus, while these tracers are informative of receptor density and occupancy, there is a critical need for development of radiotracers specifically dedicated to ovarian hormones, which could ultimately clarify how HCs modulate the delicate hormonal balance in the brain and thereby shed light on subsequent consequences for mood and depression susceptibility.

## Conclusion

In order to advance our understanding of possible effects of HC-use on mood, we propose the following three perspectives to guide future research endeavors: (1) stratification for HC methods or direct comparison of the effects of different HC methods on mood, (2) initiation and implementation of rigorous RCT designs with adequate samples based on transparent a priori power-analyses, and (3) the development of quantitative methods to differentiate between exogenous and endogenous hormonal effects.

Based on the evidence currently available, it is likely that HC-intake can lead to mood-related side effects, particularly in women with a history of previous depressive episodes. Reported data indicate a trend towards negativity bias in emotion recognition and reactivity, a trend towards a blunted reward response and a potential dysregulation of the stress response in HC-users. Of note and not extensively discussed in this review, however, are the reported positive effects of HC-use on mood in some women, especially for symptoms of PMDD (but see [20] for review). Any HC-effects on mood and the underlying psychological and neurophysiological mechanisms are therefore likely context-dependent.

It is imperative to take any reports on depressed mood as a potential side effect of HC-intake seriously given the recent reports from large cohort studies [12••, 13••] and the reality that discontinuation of HC-intake is most often motivated by such side effects, which can pose subsequent challenges in family planning [89, 90]. In general, possible mood-related HC side effects should be carefully weighed against the profound benefit of HC methods for safe family planning. A better understanding of how and when HCs affect mood is of critical importance to provide adequate contraceptive choices to women worldwide.
